# ECRG4 inhibits growth and invasiveness of squamous cell carcinoma of the head and neck *in vitro* and *in vivo*

**DOI:** 10.3892/ol.2013.1298

**Published:** 2013-04-10

**Authors:** TING XU, DAJIANG XIAO, XIN ZHANG

**Affiliations:** 1 Department of Otolaryngology, The Second People’s Hospital of Wuxi, Wuxi, Jiangsu 214002;; 2 Department of Otolaryngology, Xiangya Hospital, Central South University, Changsha, Hunan 410008, P.R. China

**Keywords:** SCCHN, ECRG4, growth, metastasis, *in vitro*, *in vivo*

## Abstract

ECRG4 has been shown to be a candidate tumor suppressor in several tumors, but its role in head and neck cancer remains poorly understood. In the present study, the effect of ECRG4 on head and neck cancer was investigated *in vitro* and *in vivo.* pFLAG-CMV-2-ECRG4 was stably transfected into squamous cell carcinoma of the head and neck (SCCHN) M2 cell lines to overexpress the ECRG4 gene. Real-time PCR and western blot analysis were performed to detect gene and protein expression, respectively. An MTT assay and flow cytometric analysis were used to detect the growth of M2 cells. Matrigel™ invasion and scratch assays were applied to observe the invasion and migration of the cells. A tumorigenicity assay was applied to test the tumor growth and cervical lymph node metastasis *in vivo*. Based on the data, pFLAG-CMV-2-ECRG4 significantly increased the expression of ECRG4 in the M2 cells. The constructed plasmid inhibited cell proliferation and promoted cell cycle arrest and apoptosis in the M2 cells. The growth rate and metastasis of the tumor cells in xenografts were suppressed following the overexpression of ECRG4 in nude mice. These data suggest that ECRG4 plays a significant role in the regulation of growth and metastasis in SCCHN, providing new clues for the diagnosis and therapy of SCCHN.

## Introduction

Squamous cell carcinoma of the head and neck (SCCHN) is one of the most common cancers that result in mortality, accounting for 6% of all cancers worldwide (1,2). The overall 5-year survival rate for patients with this type of cancer is among the lowest of the major cancer types (3). Despite the continuous improvement in surgical procedures in the past few decades, the 5-year survival rate of patients has not increased (4). One of the main reasons for this is the lack of molecular understanding with regard to head and neck cancer and the clinical benefits to patients. Therefore, a deeper understanding of carcinogenesis associated with early diagnosis and metastasis are required for the treatment of SCCHN.

Currently, the biological study focus is transitioning from the cloning of novel genes to characterizing the function of the protein product. The ECRG4 gene (GenBank accession no. AF 325503) was initially identified and cloned in the State Key Laboratory of Molecular Oncology and the Department of Etiology and Carcinogenesis in Peking Union Medical College (Peking, China) from normal human esophageal epithelium (5,6). The ECRG4 gene was first described as a novel tumor suppressor gene associated with prognosis in esophageal squamous cell carcinoma (ESCC). ECRG4 RNA or protein was used as an independent prognostic factor for ESCC (7,8). Subsequently, it was reported that ECRG4 was also involved in certain tumors, including colorectal carcinoma, prostate cancer, T-cell leukemia, gastric cancer and glioma (9–13). Epigenetic alterations in cancer include changes in the chromatin structure and in methylation of cytosine residues in the DNA (14). The head and neck epithelium is anatomically adjacent to the esophageal epithelium and although the majority of head and neck cancers are squamous carcinomas, no studies are available regarding the effects of ECRG4 on SCCHN.

Thus, the present study demonstrated the role of ECRG4 in the growth and invasiveness of SCCHN *in vitro* and *in vivo*, in order to explore new approaches for the diagnosis and treatment of SCCHN.

## Materials and methods

### Cell lines and cell culture

The SCCHN cell line, M2, is a metastatic cell line capable of generating lymph node metastasis *in vivo*. M2 cells are derivatives of Tu686 created through repeated *in vivo* selection in nude mice from a lymph node metastasis of the same patient (15). Tu686 was established from a primary tumor in the base of a human tongue. In the present study, the M2 cells were a gift from the Emory University Winship Cancer Institute, Atlanta, Georgia. The cell lines were maintained as monolayer cultures in Dulbecco’s modified Eagle’s medium (DMEM)/F12 medium (1:1) supplemented with 10% fetal bovine serum (FBS), 100 IU/ml penicillin and 100 IU/ml streptomycin at 37°C in a humidified atmosphere, with 5% CO_2_.

### Construction of eukaryotic ECRG4 expression vector and stable transfection

The ECRG4 open reading frame was amplified from cDNA clone IMAGE: 5260075 using the primers designed as follows: forward, 5′gcaagcttatggctg cctcccccgcgcg3′ and reverse, 5′gcggatccttagtagtcatcgtagttga3′. Subsequent to being digested with *Hind*III and *Bam*HI, the coding region of the ECRG4 cDNA was subcloned into the eukaryotic expression vector, pFLAG-CMV-2 (Sigma, St Louis, MO, USA). The reconstructive plasmid was named pFLAG-CMV-2-ECRG4 and was fully sequenced to ensure that no mutation was introduced during the PCR amplification. The M2 cells were transfected with pFLAG-CMV-2-ECRG4 using lipofectamine™ 2000 (Invitrogen, Carlsbad, CA, USA) according to the manufacturer’s instructions. For constructing the stable clones, cells were selected with 2000 to 3000 *μ*g/ml G418 (Calbiochem, La Jolla, CA, USA) 48 h post-transfection. The transfection efficiency was detected by RT-PCR and western blot analysis as mentioned below.

### Real-time PCR

The total RNA of the M2 cells which had undergone varying treatments were isolated by Trizol (Invitrogen, Carlsbad, CA, USA) The total RNA (1 *μ*g) was reverse transcribed by a RT-PCR kit (Toyobo Corporation, Osaka, Japan), according to the manufacturer’s instructions. The primers were designed based on previous studies (8,16) and were synthesized by Invitrogen. The primers were designed as follows: ECRG4 forward, 5′-ttccttggcagcctgaagcg-3′ and reverse, 5′-ggctccatg cctaaagccgt-3′; GAPDH forward, 5′-gtcagtggtggacctgacct-3′ and reverse, 5′-tgaggaggggagattca-3′. RT products (1 *μ*l) were amplified by PCR at 95°C for 1 min, followed by 30 cycles at 95°C for 10 sec, 60°C for 2 sec and 72°C for 30 sec and a final extension at 4°C for 5 min. The PCR product (8 *μ*l) was then electrophoresed on a 1.2% agarose gel and the intensity of the bands was quantified by FluorChem FC2 (Alpha Innotech, San Leandro, CA, USA). The PCR experiments were repeated at least three times.

### Western blot analysis

The whole cell lysates were prepared. A total of 50 *μ*g protein from each sample was mixed with gel loading buffer (2X: 125 mM Tris-HCl, pH 6.8, 4% SDS, 20% glycerol, 0.1% bromophenol blue and 2.5% β-mercaptoethanol), boiled for 5 min, separated by 8% SDS-polyacrylamide gel and then transferred onto polyvinylidene difluoride membranes. The blotted membranes were incubated with anti-ECRG4 (sc-135139, dilution 1:300), anti-cyclin A (sc-271682, dilution 1:500), anti-cyclin E (sc-25303, dilution 1:500), anti-Bax (sc-20067, dilution 1:300), anti-Bcl-2 (sc-509, dilution 1:300), anti-AKT (sc-5298, dilution 1:300), anti-p-AKT (sc-101629, dilution 1:300) and anti-E-cadherin (sc-21791, dilution 1:500) at 4°C overnight (all antibodies were from Santa Cruz Biotechnology Inc, Santa Cruz, CA, USA). Subsequent to being washed, the membranes were incubated with HRP-labeled anti-rabbit or anti-mouse for 1 h at room temperature. Bands were visualized by employing the BeyoECL Plus Detection System (Beyotime Beyotime Institute of Biotechnology, Jiangsu, China). Protein expression levels were quantified by FluorChem FC2 and represented as the densitometric ratio of the targeted protein to β-actin. Cell protein lysates were assayed in triplicate.

### Cell proliferation assays

An MTT assay was employed to detect the cell proliferations. The transfected cells were seeded onto 96-well plates at a density of 2.5×10^3^ cells/well. The medium was replaced with fresh medium containing 5 mg/ml MTT reagent (Sigma, St. Louis, MO, USA) following various durations of culturing (a total of 7 days). The cells were cultured for another 4 h at 37°C, then 100 *μ*l DMSO was added to each well and mixed vigorously to solubilize the colored crystals produced within the living cells. The absorbance at 490 nm was measured using a BIO-TEK microplate reader (Bio-Rad, Hercules, CA, USA). Each experiment was repeated in triplicate.

### Flow cytometric analysis of cell cycle arrest and apoptosis

The transfected cells were seeded onto a 6-well plate at a density of 10^5^ cells per well in RPMI-(DMEM)/F12 medium for 48 h. For the cell cycle analysis, following incubation, the cells were harvested and fixed in ice-cold 70% (v/v) ethanol for 15 min. The cells were then treated with RNase A and stained with 50 *μ*g/ml propidium iodide (PI), followed by incubation at 37°C for 30 min in the dark. Samples were analyzed by a FACScan flow cytometer (Becton Dickinson, Franklin Lakes, NJ, USA), according to the manufacturer’s instructions. The DNA content in the G_1_, S and G/M phases was analyzed using BD FACSDiva™ software (Becton Dickinson). The analysis of apoptosis was performed using an ApopNexin™ FITC Apoptosis Detection kit (Millipore, Lake Placid, NY, USA). Briefly, the cells were collected and washed twice with cold PBS and re-suspended in 200 ml 1X binding buffer, prior to 10 *μ*l annexin V-FITC and 20 *μ*g/ml PI being added. Following gentle vortex mixing, the cells were incubated in the dark for another 20 min at room temperature. The samples were analyzed by a FACScanto™ II flow cytometer (Becton Dickinson). All experiments were performed in triplicate.

### Matrigel™ invasion and scratch assays

The cells were seeded at 2.5×10^4^ cells per well on Matrigel coated inserts (8 *μ*m pores; BD Bioscience) in serum free medium. Following a 48 h incubation, the cells which penetrated the filters were stained with gentian violet. The number of invasive cells was determined by counting all cells attached to the bottom of the inserts under an inverted microscope at ×10 magnification. All experiments were conducted independently in triplicate.

For the scratch assay, each well of the 6-well plates was marked with five straight black lines. The cells were seeded onto the plates for 12 h in complete medium (5×10^5^ cells per well). Scratch wounds were applied in each well with a 200-*μ*l pipette tip and the non-adherent cells were washed off with medium. Fresh serum free medium was added to the wells and the cells were incubated for ≤48 h. Inverted microscope images were captured at 0 and 48 h subsequent to scratching. All experiments were conducted independently in triplicate.

### Nude mouse experiments

Male, 5-week-old BALB/c nude mice were purchased from the Institute of Laboratory Animal Sciences (Beijing, China). The mice were then observed daily for their diet consumption, stools and mental state, and the tumor size and body weight were measured every three days. A total of 50 ml cell solution from the varying groups (control, vector and ECRG4, each containing 2.5×10^6^ tumor cells) were injected into the mylohyoid muscle of three groups of mice (each containing 8 mice) two weeks after arrival. The lengths and widths of the tumors were measured with Vernier calipers and calculated using the following formula: Tumor volume = length × width^2^ × 0.5. The mice were sacrificed 25 days later in accordance with institutional regulations for animal experiments. The use of animals in the present study complies with the Guide for the Care and Use of laboratory Animals. The study was approved by the Institutional Animal Care and Use Committee, Wuxi, Jiangsu, China.

### Statistical analysis

The SPSS 17.0 for Windows statistical analysis software package (SPSS, Inc., Chicago, IL, USA) was employed for the analysis of the data. The Student’s t-test and Mann-Whitney U test were used for the statistical analysis of the data. P<0.05 was considered to indicate a statistically significant difference.

## Results

### Overexpression of ECRG4 in SCCHN cell lines following stable transfection

**Figure 1:**
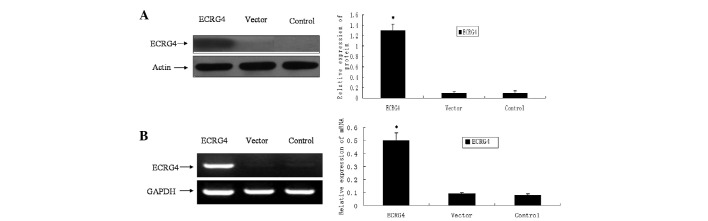
Overexpression of ECRG4 in M2 cells. The reconstructive plasmid pFLAG-CMV-2-ECRG4 was stably transfected into the M2 cells. Real-time PCR and western blot analysis were applied to detect the transfection efficiency. Briefly, the cells without interference or those cells transfected with pFLAG-CMV-2 and pFLAG-CMV-2-ECRG4 were referred to as the control, vector and ECRG4, respectively. (A) Increased protein expression of ECRG4 detected by western blot analysis. (B) Increased mRNA expression of ECRG4 as revealed by RT-PCR. ^*^P<0.05 vs. control group.

pFLAG-CMV-2-ECRG4 was stably transfected into M2 cells to upregulate the expression of the ECRG4 gene. The control or vector consisted of cells without interference or those transfected with pFLAG-CMV-2, respectively. As shown in Fig 1, the selected clones were able to overexpress ECRG4. In the control and vector cells, the ECRG4 mRNA and protein were minimally expressed in the cells. However, the two were significantly upregulated following transfection with pFLAG-CMV-2-ECRG4 (Fig. 1).

### Overexpression of ECRG4 inhibits cell proliferation of M2 cells in vitro

**Figure 2:**
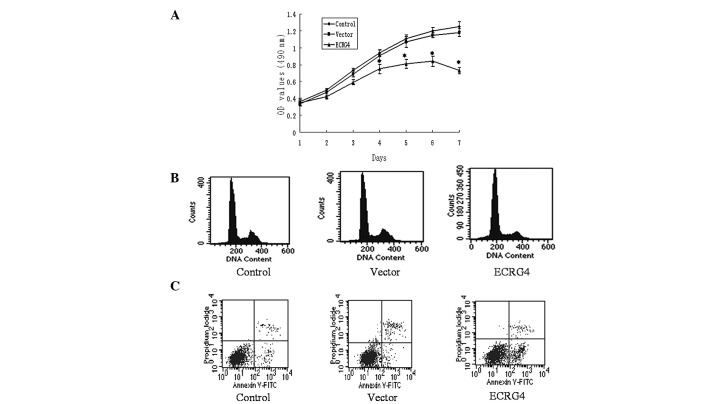
Effects of ECRG4 on cell growth and apoptosis of M2 cells. The cells without interference or those transfected with pFLAG-CMV-2 and pFLAG-CMV-2-ECRG4 were referred to as the control, vector and ECRG4, respectively. (A) Cell proliferation was detected by MTT assay every 24 h for a total of 7 days. Results represent the mean ± SD of 3 independent experiments (^*^P<0.05 vs. control group). Flow cytometric analysis of (B) cell cycle arrest and (C) apoptosis. The percentage of cells in the ECRG4 group in the G_0_/G_1_ phase was significantly higher than those of the control and vector groups (P<0.05 vs. control group). The apoptotic rate of the cells was significantly increased (P<0.05 vs. control group). OD, optical density.

In order to reveal the effect of the ECRG4 gene on SCCHN cell proliferation *in vitro*, an MTT assay was applied to the M2 cells and the growth curve was obtained. As presented in Fig. 2A, the cells of the experimental group grew significantly slower than the cells of the control and vector groups. This indicates that ECRG4 may inhibit the proliferation of SCCHN cells.

### Overexpression of ECRG4 induces cell cycle arrest in G0/G1 phase and promotes apoptosis in M2 cells

To further demonstrate the mechanism of ECRG4-induced cell growth inhibition in the M2 cells, flow cytometry was performed to examine the cell cycle and apoptosis. The cytometric analysis showed that the percentage of G_0_/G_1_ phase cells in the experimental group was 76.74±3.22, which was significantly more than those identified in the control and vector groups (59.31±2.04 and 61.49±1.82%, respectively; P<0.05). The apoptotic rate of the cells significantly increased post-transfection (16.30±4.36 vs. 4.07±4.70 and 3.91±3.59%; P<0.05; Fig. 2B and C).

### Effect of ECRG4 gene on cell cycle and apoptosis-related proteins

**Figure 3:**
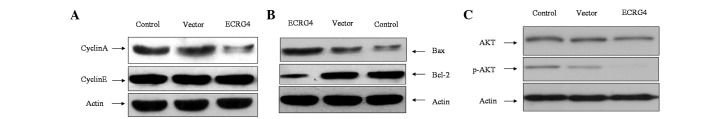
Effect of ECRG4 on cell cycle- and apoptosis-related proteins. (A) Cyclin A expression significantly decreased, while there was no significant difference in Cyclin E expression. (B) ECRG4 increased the Bax expression and decreased the Bcl-2 expression. (C) Overexpression of ECRG4 resulted in the inhibition of phosphorylation of AKT in the M2 cells. The results are of one representative experiment among three runs that showed similar patterns with one another.

To investigate the mechanism involved in ECRG4 gene-induced G_0_/G_1_ cell cycle arrest, the cyclins were examined. As shown in Fig. 3A, Cyclin A expression decreased significantly, while there was no difference in the expression of Cyclin E. To investigate the potential mechanism involved in ECRG4-induced apoptosis, the expression of Bax and Bcl-2 was detected. The results showed that following transfection, Bax expression was significantly increased and Bcl-2 expression was decreased (Fig. 3B).

### ECRG4 inhibits phosphorylation of AKT

To further investigate the potential molecular mechanism involved in ECRG4-induced cell growth inhibition, the expression of p-AKT was examined. It was shown that AKT phosphorylation was significantly inhibited, which suggests that ECRG4 may be involved in the PI3K/AKT pathway in SCCHN (Fig. 3C).

### ECRG4 inhibits cell invasiveness and migration in M2 cells

**Figure 4:**
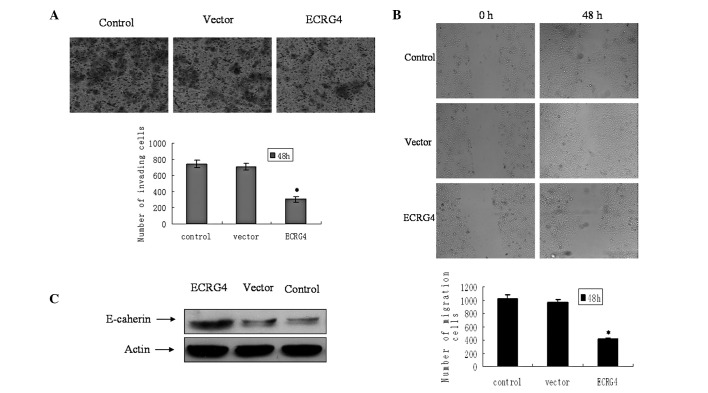
Effect of ECRG4 on the invasion and migration of M2 cells *in vitro*. (A) Images were captured using inverted microscopy for the cells which penetrated through the filters to the other side of inserts following various treatments. (B) Images were captured for the wound closure of cells at 0 and 48 h following treatment. Graphs represent the average number of migrating cells from five independent fields. (C) Expression of epithelial marker-E-cadherin in the M2 cells increased significantly following transfection with ECRG4. Error bars represent the standard error of the mean. (^*^P<0.05).

The effect of ECRG4 on cell invasion was measured in the M2 cells. The cells which penetrated through the filters to the other side of the inserts in the experimental group were fewer in number compared with those in the control and vector groups (Fig. 4A). The capability for migration following transfection was then evaluated. The cells transfected with ECRG4 showed lower motility and achieved less wound closure at 48 h (Fig. 4B). The epithelial marker E-cadherin was also significantly upregulated (Fig. 3C).

### Overexpression of ECRG4 suppresses growth and metastasis of xenografts in nude mice

**Figure 5:**
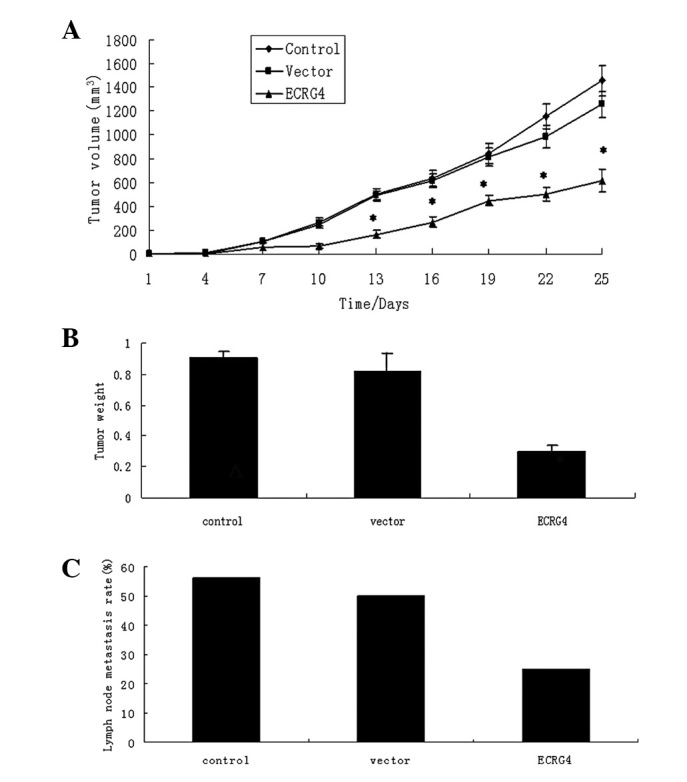
Effect of EGCR4 on the growth and metastasis of SCCHN *in vivo*. Groups of nude mice were inoculated with 2.5×10^6^ M2 cells that had undergone different treatments. Vector, transfected with pFLAG-CMV-2; ECRG4, transfected with pFLAG-CMV-2-ECRG4. The development of solid SCCHN tumors was monitored every 3 days. The mice were sacrificed 25 days post-inoculation and their tumor weights were measured. (A) The curves of SCCHN tumor volume. (B) The quantitative measurement of tumor weights. Data are expressed as mean ± SD of each group. (C) Comparison of numbers of cervical lymph node metastasis.

The M2 cells were applied to the *in vivo* experiment due to their high invasiveness. The three groups of cells (control, vector and ECRG4) were injected into the mylohyoid muscle of the nude mice and the tumor formation was carefully observed. The tumor weights and volumes were measured at 25 days following cell injection. The growth rate of the ECRG4-overexpressed tumors was significantly decreased. The tumor volumes and weights of the mice in the experimental group were significantly less than those of the control and vector tumors (P<0.05). However, no significant difference was detected in either the tumor volume or weight between the control and vector groups (both P>0.05; Fig. 5). Bilateral and unilateral cervical lymph node metastasis was exhibited by only one and three mice, respectively, in the ECRG4 group (lymph node metastasis rate 4/16; 25%); by two and four mice, respectively, in the vector group (lymph node metastasis rate, 8/16; 50%); and by three and three mice, respectively, in the control group (lymph node metastasis rate, 9/16; 56.25%). The metastasis rate of the ECRG4 group was significantly lower than that of the control and vector groups (both P<0.05), while there was no significant difference between the control and vector groups (P>0.05; Fig. 5). No lung metastasis was identified from serial sections in any of the groups. These data indicate that the overexpression of ECRG4 decreases cervical lymph node metastasis *in vivo*.

## Discussion

Recent studies on a novel gene, ECRG4, in other types of carcinomas (8–13) have attracted attention to its potential role in SCCHN. The present study demonstrated the effect of the ECRG4 gene on the growth and metastasis of SCCHN *in vitro* and *in vivo,* and revealed a prognostic marker that contributes to the malignant properties of SCCHN. To the best of our knowledge, this was the first study associated with the mechanism of function for ECRG4 in SCCHN.

pFLAG-CMV-2-ECRG4 successfully upregulated the expression of ECRG4 in the M2 cells. Cell cycle arrest and the induction of apoptosis are major mechanisms involved in anti-cancer treatments (17). The present study showed that ECRG4 inhibited cell growth and induced G_0_/G_1_ cycle arrest and apoptosis in the M2 cell lines. This was consistent with the study by Li *et al* with regard to ECRG4-induced cell cycle arrest in esophageal carcinoma (18). Although other studies have investigated the process of cell cycle arrest and apoptosis in other cancers, the analysis of the exact mechanism was not thorough (6–14). Cell cycle progression is tightly regulated by cyclin/CDK complexes. The kinase activity of CDKs is modulated by their regulatory subunits known as cyclins. The expression level of the cyclins is also an important determinant in cell cycle progression, particularly during G_1_/S and G_2_/M transitions (19). In the present study, ECRG4 decreased the expression of cyclin A but not cyclin E, indicating that cyclin A may play a key role in ECRG4-induced cancer cell arrest at the G_0_/G_1_ cell cycle phase. Bcl-2 family members appear to play a significant role in the regulation of the intrinsic pathway of apoptosis (14). Changes in the ratio of Bcl-2 and Bax proteins in mitochondria may cause a loss of membrane potential, the release of cytochrome-*c* and the activation of caspase-9 (20). Li *et al* demonstrated that ECRG4 inhibits glioma proliferation and induces cell apoptosis through the NF-κb signaling pathway (18). Matsuzaki *et al* revealed that ECRG4 is a novel antiapoptotic gene involved in the negative regulation of caspase-8-mediated apoptosis in T-leukemia cells (12). However, the present study identified a novel signaling pathway for ECRG4-induced apoptosis. The results showed that ECRG4 increased the expression of Bax and decreased the expression of Bcl-2 in the M2 cell line, which revealed that ECRG4 may trigger the mitochondrial pathway of apoptosis through Bax/Bcl-2. Disruption of normal PI3K signaling has been documented as a frequent occurrence in several human cancers and appears to play a significant role in their progression (21,22). The suppressed phosphorylation of AKT may provide a mechanism to explain the role of ECRG4 in controlling SCCHN cell proliferation.

Cell invasion and migration play significant roles in cancer metastasis. SCCHN has a characteristically high rate of relapse and a high facility for metastasis (23). The present study showed that ECRG4 was able to suppress SCCHN cell invasion and migration, implicating its potential involvement in cancer metastasis. This observation was consistent with a study on gliomas in which ECRG4 was observed to reduce cell invasion (18). Epithelial-mesenchymal transition (EMT) has previously been implicated to be a key mechanism involved in cancer metastasis (23). A hallmark of EMT is the loss of E-cadherin expression. Our preliminary experiment (24) showed that as a high metastatic derivative of Tu686, M2 cells demonstrate a lower expression of E-cadherin. In the present study, this was reversed following the transfection with ECRG4. This indicates that ECRG4 may suppress the invasion of SCCHN cells by reversing the progress of EMT.

Although numerous studies (6–14) have focused on the effects of ECRG4 in other cancers using the clinical and *in vitro* levels, studies on animals are rare. The present *in vivo* study further demonstrated that ECRG4 was able to markedly diminish the tumorigenicity of SCCHN. Submandibular injections of SCCHN M2 cells were successfully able to induce lymph node metastasis, while injections of pFLAG-CMV-2-ECRG4-interfered cells caused apparent inhibition of cervical lymph node metastasis. These results may profoundly demonstrate a critical role for ECRG4 in SCCHN cancer, thus providing strong evidence for a clinical diagnosis and treatment.

To the best of our knowledge, this is the first study to demonstrate the effect of the ECRG4 gene on the growth and metastasis of SCCHN *in vitro* and *in vivo*. The results revealed that ECRG4 gene expression results in the suppression of the growth rate and metastasis of SCCHN tumors. Increasing evidence indicates that ECRG4 inhibits cell growth and invasion in other human malignancies, however, the molecular mechanisms involved require further research.
